# Reply to: Critical evaluation of (110) texture in lithium electrodeposits on isotropic Cu polycrystals

**DOI:** 10.1038/s41467-022-32950-5

**Published:** 2022-09-30

**Authors:** Qing Zhao, Jingxu Zheng, Lynden A. Archer

**Affiliations:** 1 Robert Frederick Smith School of Chemical and Biomolecular Engineering, Cornell University, Ithaca, NY 14853 USA; 2 Department of Materials Science and Engineering, Cornell University, Ithaca, NY 14853 USA; 3 Department of Physics, Massachusetts Institute of Technology, Cambridge, MA 02139 USA

**Keywords:** Batteries, Batteries

**replying to** C. Lu et al. *Nature Communications* 10.1038/s41467-022-32949-y (2022)

In Lu et al.’s comment, the possibility of achieving textured crystal growth on textureless polycrystalline substrates is questioned. As stated in Lu et al.’s comment, their doubt arises mainly from two claims: (1) the unlikelihood of growing a textured film on textureless substrates, e.g., “It is hard to imagine that the Li electrodeposits of dominant 110 texture could be grown on polycrystalline Cu foil;” and (2) methodological errors associated with use of θ–2θ scan to identify material textures, e.g., “The crystallographic texture (preferred orientation) was determined by θ–2θ scan x-ray diffraction (XRD).” Unfortunately, both claims reflect misunderstanding of the complexities of crystal growth associated with electroreduction reactions in an electrochemical cell.

We first address the question about the possibility of growing textured films on textureless substrates. There is in fact a large body of readily available literature (an abbreviated list is provided in Table 1), which reveals textured electrodeposition on textureless substrates^1–4^. A better question then might be the reverse of the question posed by Lu et al.: why are many electrodeposited metal films textured, even when deposition occurs on textureless substrates? The answer to this latter question requires precise understanding of how crystalline metals nucleate and grow at interfaces in an electrochemical cell. Crystal growth of metal deposits in electrochemical cells typically occurs at a planar/nearly-planar electronically conducting substrate in a multi-step sequence: transport of solvated metal ions to the interface; desolvation of the ions; and electroreduction to form the metal deposit. Importantly, each of these processes occur in anisotropic chemical potential, electric, and stress (via the polymeric separator) fields in the electrochemical cell. It is now well received knowledge that growing crystals in anisotropic fields drives development of textures in order to lower the total energy. There is in fact a rather large, existing body of work showing how anisotropic fields of a variety of natures, e.g., magnetic, electric, stress, chemical potential, etc., drives electrochemical crystal growth in preferred orientations, in the absence of any texturing of the substrate (see Table 1). Some of these studies in fact show that the texture of an electrodeposited metal film can be effectively tuned by manipulating the deposition protocol, e.g., pulsing^5,6^. There are of course exceptions, where loss of texturing of metal electrodeposits in anisotropic fields can be caused by electrokinetic instability. For example, in dilute electrolytes characterized by depletion layer thicknesses much larger than the largest dimension of the crystal, large body forces produced by the electric field gradient can cause localized swirling hydrodynamic flows (i.e., electroconvection), which produces texture loss. Heterogeneities (e.g., non-uniform surface passivation of reactive metal deposits) can also result in loss of texture of metal electrodeposits^7–9^. On these bases, we therefore conclude that even a cursory reading of the large body of published experimental work and minimal understanding of the fundamental anisotropic nature of the fields in an electrochemical cell, one would reach a conclusion exactly opposite to Lu et al., namely, that textured crystal growth on textureless substrates is more of the norm in an electrochemical cell.

**Table 1:** Representative literature reports on deposition textures and their characterization using θ–2θ scans

Year	Methods	Material	Substrate	Harris’s method /Lotgering factor	DOI
1987	Electrodeposition	Nb	Cu	Yes	10.1007/BF01023291
2000	Electrodeposition	Ni	Cu	Yes	10.1016/s0041-624x(99)00215-2
2000	Electrodeposition	Ni	Brass	Yes	10.1016/S0254-0584(00)00313-8
2001	Electrodeposition	Zn	Steel	Yes	10.1080/00202967.2001.11871375
2005	Electrodeposition	TiB_2_	Graphite	Yes	10.1016/j.matlet.2005.05.050
2005	Electrodeposition	Fe-Ni alloy	Steel	Yes	10.1016/j.surfcoat.2004.11.035
2005	Electrodeposition	In doped ZnO	Cu	Yes	10.1016/j.tsf.2005.04.042
2006	Electrodeposition	Zn	Steel	Yes	10.1016/j.surfcoat.2005.12.036
2007	Electrodeposition	Au and Cu wires	Cu	Yes	10.1088/0957-4484/18/13/135709
2007	Electrodeposition	Ni-based composites	Steel	Yes	10.1016/j.surfcoat.2007.12.005
2007	Electrodeposition	Zn-Co alloy	Steel	Yes	10.1016/j.surfcoat.2007.07.023
2007	Electrodeposition	Cu	Ni-P alloy	Yes	10.1016/j.surfcoat.2007.02.011
2010	Electrodeposition	Fe doped CdSe	ITO	Yes	10.1016/j.mseb.2010.03.054
2010	Electrodeposition	ZnO	FTO	Yes	10.1021/jp9087145
2011	Electrodeposition	Zn	Steel	Yes	10.1016/j.jelechem.2011.10.001
2011	Electrodeposition	Ni-based composites	Steel	Yes	10.1016/j.surfcoat.2011.02.057
2011	Electrodeposition	Cu	SiO_2_ coated Si	Yes	10.1021/cg200877f
2011	Electrodeposition	Zn	Steel	Yes	10.1007/s10800-010-0205-8
2011	Electrodeposition	Al	W-Cu alloy	Yes	10.1016/j.surfcoat.2011.03.058
2012	Electrodeposition	CdSe	ITO	Yes	10.1016/j.mssp.2011.10.007
2013	Electrodeposition	Zn	Steel	Yes	10.1007/s10800-012-0518-x
2013	Electrodeposition	Ni	Cu	Yes	10.1016/j.apsusc.2013.12.053
2014	Electrodeposition	Ni-CeO_2_	Steel	Yes	10.1016/j.matchemphys.2014.06.057
2014	Electrodeposition	Ni-Al	Steel	Yes	10.1016/j.jallcom.2014.03.063
2014	Electrodeposition	Cu	Cu	Yes	10.1016/j.cattod.2014.08.008
2015	Electrodeposition	W	Mo	Yes	10.1016/j.apsusc.2014.11.186
2015	Electrodeposition	Ni	Pd coated Cu	Yes	10.1149/2.0381507jes
2016	Electrodeposition	Ni	Cu	Yes	10.15344/2455-2372/2016/123
2017	Electrodeposition	In	Cu	Yes	10.1016/j.electacta.2017.03.082
2018	Electrodeposition	Zn	Glassy carbon	Yes	10.1016/j.hydromet.2017.10.030
2019	Electrodeposition	Co	Cu	Yes	10.1016/j.matchemphys.2019.122395
2019	Electrodeposition	Cu deoped SnS	ITO	Yes	10.1007/s10854-019-01924-7
2020	Electrodeposition	Zn-Co alloy	Steel	Yes	10.1080/00202967.2020.1748390
2020	Electrodeposition	Zn	Cu	Yes	10.1007/s11581-019-03293-x
2020	Electrodeposition	Co	Cu	Yes	10.1016/j.matchemphys.2019.122395
2021	Electrodeposition	Sn	Ni	Yes	10.1007/s10008-020-04894-7
2021	Electrodeposition	Bi	Cu	Yes	10.1016/j.jallcom.2021.161451
1975	Vapor deposition	TiN	Fe	Yes	10.1149/1.2133409
2003	Vapor deposition	TiC/TiB_2_	WC	Yes	10.1016/S0257-8972(02)00666-7
1988	Vapor deposition	PbTiO_3_	Ti	Yes	10.1149/1.2095517
1991	Vapor deposition	Diamond	Si	Yes	10.1016/0257-8972(91)90302-D
1992	Vapor deposition	Al doped ZnO	Glass	Yes	10.1063/1.351309
1995	Vapor deposition	SiC	Graphite	Yes	10.1016/0040-6090(96)80023-x
1995	Vapor deposition	CdTe	ITO	Yes	10.1116/1.579607
1996	Vapor deposition	CrN	Steel	Yes	10.1016/s0257-8972(96)03071-x
1997	Vapor deposition	RuO_2_	SiO_2_ coated Si	Yes	10.1149/1.1837530
2009	Vapor deposition	TiO_2_	Quartz and Si	Yes	10.1021/cg9001779
2009	Vapor deposition	TaC	Graphite 002	Yes	10.1016/j.tsf.2008.11.058
2011	Vapor deposition	Ga doped ZnO	Glass	Yes	10.1016/j.optmat.2010.12.008
2011	Vapor deposition	TaC	Graphite 002	Yes	10.1016/j.apsusc.2010.11.172
2012	Vapor deposition	SiC	C	Yes	10.1111/j.1744-7402.2012.02786.x
2014	Vapor deposition	ZnO	Glass	Yes	10.1016/j.tsf.2014.06.033
2016	Vapor deposition	AuN	Steel	Yes	10.1016/j.surfcoat.2016.11.081
2019	Vapor deposition	TiN	Steel, WC, etc.	Yes	10.1039/c9ce00488b
2020	Vapor deposition	Ga doped ZnO	SiO_2_ coated glass	Yes	10.1038/s41598-020-57532-7
2020	Vapor deposition	In_2_O_3_	Glass	Yes	10.1016/j.mssp.2020.105195
2021	Vapor deposition	Diamond	WC-Co	Yes	10.1016/j.ceramint.2020.10.124

Next, we consider the second aspect of the comment by Lu, et al. concerning limitations in using θ–2θ scans to identify out-of-plane texture. We again would first simply draw the readers’ attention to the large body of contemporary literature which uses θ–2θ scans as a convenient, yet powerful tool to characterizea materials’ texture^10,11^. One might rightly ask whether the convenience of the method comes at the expense of accuracy. To address this point we consider the original Harris paper, “Quantitative Measurement of Preferred Orientation in Rolled Uranium Bars”, published in 1952, where the method was first disclosed^12^. Sometimes called the “Harris method/coefficient” or simply “θ–2θ scan”, the method has been discussed, examined, and evaluated over the decades. Engler et al., for example, noted in their classic textbook on texture analysis: “inverse pole figures can also directly be measured by means of diffraction methods (citing the Harris paper and a few others)… in a θ–2θ scan^13^.” More importantly, Peterson et al. experimentally verified using Pb(Zr_0.6_Ti_0.4_)O_3_ ferroelectric films as an example that “reasonable agreement” is obtained in comparisons of the Harris method and 2D pole figures^14^. Due to the technical convenience and the fact that fields near a substrate (e.g., an electrode in an electrochemical cell) oftentimes develop an out-of-plane, uniaxial symmetry parallel to the normal direction, θ–2θ scan have in fact been consistently used in the field of electrodeposition/electrometallurgy^15,16^, as well as in other fields^17,18^, to characterize textures. An abbreviated list of these studies is provided in Table 1.

So why are the θ–2θ scans so useful? To a first order approximation, the integrated X-ray diffraction intensity of a given peak of a textureless material (e.g., powder) in a θ–2θ scan can be calculated as ${I}_{{hkl}}\propto {\left|{S}_{{hkl}}\right|}^{2}{\cdot M}_{{hkl}}\cdot {Lp}(\theta )\cdot {e}^{-B{(\frac{{\sin }\theta }{\lambda })}^{2}}$, where *S* is the structure factor, *M* is the multiplicity, *Lp*(*θ*) is the Lorentz-polarization factor and ${e}^{-B{(\frac{{\sin }\theta }{\lambda })}^{2}}$ is a temperature-dependent factor. In a θ–2θ scan, however, only crystallites with (hkl) planes aligned roughly parallel to the film surface will satisfy the kinematic diffraction condition and contribute to the experimentally measured pattern (see Fig. 1). For a given crystal structure, the only possibility for the relative peak intensity ratio in a θ–2θ scan to deviate *markedly* from this result (e.g., intensification and absence of certain peaks) is to form textures^19^. For example, in the extreme case of single crystal or singly-oriented crystallites with (hkl) parallel to the film surface, only the (hkl)-family reflections can be detected. By comparing the measured diffraction pattern to the textureless powder’s pattern (i.e., calculating the texture coefficient ${p}_{{h}_{1}{k}_{1}{l}_{1}}=\frac{{I}_{{h}_{1}{k}_{1}{l}_{1}}}{\sum {I}_{{hkl}}}/\frac{{{I}^{{\prime} }}_{{h}_{1}{k}_{1}{l}_{1}}}{\sum {{I}^{{\prime} }}_{{hkl}}}$), one is able to assess if the material is textured, and if so, which planes are preferentially aligned with the film surface and to what extent^12^.

**Fig. 1:**
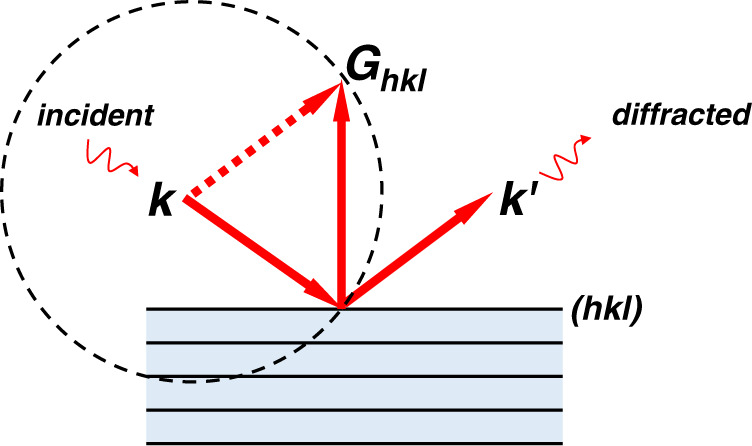
Illustration of Laue condition in a θ–2θ geometry. Only (hkl) planes perpendicular to the surface normal of the sample—with their corresponding reciprocal lattice vectors ***G***_***hkl***_ pointing out-of-plane— are able to generate a detectable diffracted X-ray in θ–2θ symmetric scans. ***k*** and ***k’*** are the wave vectors of the incident and the diffracted X-ray beams, respectively. The Ewald sphere is depicted by the dashed circle.

A perhaps obvious caveat is that this analysis assumes the texture does not possess a significant off-ND component; θ–2θ obviously cannot capture texturing with complex off-ND components since it only measures diffraction from the crystallites parallel to the film surface. For example, θ–2θ cannot capture the orientational order of a zero-background plate made of intentionally miscut quartz or Si single crystal^20,21^. This possibility, however, is clearly ruled out by the 2D diffraction pattern in Fig. 2a of our original paper, which Lu et al. do not appear to notice. Therefore, the strong intensification of the (110)_Li_ peaks and the significant weakening/absence of other Li peaks in the XRD patterns shown in Fig. S3 of the original paper—which were collected using a point detector in symmetric θ–2θ scans—reflect the existence of a significant preferred orientation in the electrodeposited Li films.

Instead, Lu et al. attribute the absence of (200)_Li_ to the change of irradiated volume as the incident angle increases, and cited Tie et al.’s data on Zn_4_SO_4_(OH)_6_·5H_2_O for support. We were initially intrigued by this point and studied the Zn_4_SO_4_(OH)_6_·5H_2_O results. According to Lu et al.’s argument based on the decreasing irradiated volumes at higher incident angles, the diffraction intensity should exhibit a downward shift; that is, the intensity ratio between (002) and (003) should be greater than 100:77 measured in a textureless powder sample. The experimental results presented by Tie et al. can be straightforwardly shown to directly contradict the very claim made by Lu et al.: the (003) peak intensity is in fact even higher than the (002) peak intensity (e.g., see Fig. S11 in Tie et al.’s paper^22^). The authors also regrettably do not appear to notice that the electrodeposited Li in our work is thicker than 50 microns. The change in irradiated volume may introduce a slopy background or a small deviation from the theoretical intensity ratio to the diffraction pattern, but can by no means suppress the emergence of a major peak, e.g., (200)_Li_ in Fig. S3 in our paper. More importantly, the incident beam is fixed at a constant angle when collecting each 2D XRD pattern, resulting in a constant irradiated volume. This simple fact evidently invalidates Lu et al.’s statements that attribute the weakening of (200)_Li_ in 2D XRD patterns to the changes in the irradiated volume.

In somewhat more rigorous terms, one might contend that the sample could have out-of-plane fiber textures of more than one Li planes—e.g., (110)_Li_ and (200)_Li_, etc.—at the same time from different crystallites. Fixing the incident X-ray at the Bragg angle of (110)_Li_, diffraction from other planes, e.g., (200)_Li_, will not be triggered since the Laue condition is not satisfied for those otherwise fiber-textured crystallites. The θ–2θ scans shown in Fig. S3, however, evidently do not support this conjecture because the existence of the additional fiber textures would produce pronounced diffraction with the symmetric setup used for the experiments. Specifically, in this set-up the incident angle is varied within a large range capturing the Bragg angles of the major peaks of Li. We’ve therefore invalidated all possibilities, but that the Li films in question have (110) out-of-plane preferred orientation.

We note further that Lu et al.’s comment that the (211)_Li_ ring is beyond the 2D detector’s measurement range is also incorrect. The range of interest selected for the 2D XRD characterization is from ~33.4° to ~66.2°, which covers all the major peaks of lithium and copper, including (110)_Li_ at ~36.3°, (111)_Cu_ at ~43.4°, (200)_Cu_ at ~50.5° (200)_Li_ at ~52.3°, (211)_Li_ at ~65.1°. In Fig. 2D of the original paper, the readers can clearly see the strong (111), (200) peak of Cu, (110) peak of Li, and very weak diffraction of (200)_Li_ and (211)_Li_. The color-contour scale is fair for all the samples.

Finally, we address a number of perhaps minor, but again problematic statements made by Lu et al. The authors state, for example, that texture is the “professional term” but preferred orientation, crystal orientation, etc. are not. This view is in fact quite incorrect and contradicted by the literature^19,23^. Additionally, Lu et al. analyze the 2D patterns and conclude Li is textureless based on the absence of “strong spots” within a small *χ* angle of the Debye-Scherrer rings limited by the area of the detector. Strong spots are generated by single crystals or singly-oriented crystallites; significant mosaicity can obviously exist for textured materials^23^. Lu et al. also referenced our recent work on deformation-induced textures and invoked similar arguments against the texture identification^24^. Interestingly, Fig. 1 of ref. 24 demonstrates a good consistency between X-ray diffraction and atomic-resolution transmission electron microscopy (TEM), which again directly challenges Lu et al.’s assertions. Beyond the phenomenology, it is as important to point out that these observations are consistent with theories on the mechanisms of metal deformation. Indeed, as authors of ref. 25, we concur with Lu et al. that TEM—particularly under cryogenic conditions—is becoming a powerful tool to characterize the spatial, chemical and crystallographic information of metal anodes^25^. Unfortunately, a careful reading of that paper would again show (quite unsurprisingly) that Li tends to expose (110) plane and develop (110) texture upon deposition^26,27^. To conclude, we point out to the readers that a broader context and library of relevant resources about texturing of battery electrodes, as well as about nuances associated with texture characterization, is available in a recent review^28^.

## Data Availability

No new data were generated for the reply.
